# Editorial: Changes in the Journal

**Published:** 1970-01

**Authors:** 


### EDITORIAL

### CHANGES IN THE JOURNAL

This number of the Journal comes in a new size and shape and with a new cover. This is no mere gimmick. The change in size, together with the double column arrangement of the text, will achieve some economy in production costs and should prove more attractive to advertisers by providing a better lay-out for their displays. While we do not intend to let the advertisement pages outnumber those of text, we hope that these measures will ease, to some extent, the burden on the Society that the Journal has become and which is referred to by our retiring Treasurer elsewhere in this number. We are eternally grateful to the Society for its continued support of what is believed to be the oldest provincial medical journal, with a record of continuous publication, in Britain today, and would like to make some effort in return.

The opportunity has been taken to change the cover design. The background impression of the "Royal Fort" building of the University in which the Society's meetings were held for so many years no longer seemed appropriate, as the Society now meets in the new Medical School. The latter, although a fine building in many ways, proved rather difficult to depict recognisably in a few lines. We have therefore selected a design which local readers will recognise as an impression of the coat of arms of the City and County of Bristol.

It is with regret that we have to record that owing to increasing commitments, Dr. A. B. Raper, Joint Editor for the last eight years has felt himself obliged to give up the position. The Journal and the Society are deeply indebted to him for an immense amount of hard work and his experience, enthusiasm and solid reliability will be greatly missed.

The enlarged editorial committee appointed eighteen months ago has suggested several innovations which we hope to introduce, together with the revival of some former features which have been absent from the Journal for some years. But if the Journal is to justify the generosity of the Society in keeping it alive and if it is to continue to reflect medical thought and practice in Bristol and the West of England, it needs the help of more than an editorial committee; it needs the help of the whole Society in finding material to publish. May we once again commend to our readers the appeal, printed in the front of every number, for articles, notes and news items of all descriptions. If such assistance is freely and readily forthcoming the Journal can attempt to be worthy of the Society which gave it birth.

